# Experimental Study of Fatigue and Fracture Behavior of Carbon Fiber-Reinforced Polymer (CFRP) Straps

**DOI:** 10.3390/polym14102129

**Published:** 2022-05-23

**Authors:** Jing Gao, Penghai Xu, Lingyun Fan, Jinfeng Li, Giovanni Pietro Terrasi, Urs Meier

**Affiliations:** 1 Department of Civil Engineering, Xiamen University, Daxue Road 182, Xiamen 361005, China; 25320211152338@stu.xmu.edu.cn (P.X.); fly950920@163.com (L.F.); l347218244@163.com (J.L.); 2 Mechanical Systems Engineering Laboratory, Empa, Swiss Federal Laboratories for Materials Science and Technology, Ueberlandstrasse 129, 8600 Duebendorf, Switzerland; giovanni.terrasi@empa.ch (G.P.T.); urs.meier@empa.ch (U.M.)

**Keywords:** CFRP strap, fracture behavior, fatigue behavior, temperature effect

## Abstract

The hanger is one of the important components for through and half-through arch bridges. Conventional steel hangers are vulnerable to corrosion due to corrosive environments. Therefore, a new type of bridge hangers consisting of Carbon Fiber-Reinforced Polymer (CFRP) straps was developed recently. The CFRP straps are self-anchored, which is formed by layers-winding, and they have great advantages in corrosive environments such as high resistance to corrosion. In this study, the fatigue and fracture behavior of CFRP straps has been experimentally investigated. Firstly, the tensile testing of four CFRP strap specimens was conducted to investigate the static fracture behavior of CFRP straps, and three stages were observed, including delamination, cracking, and brittle rupture. Then, a fatigue test of thirty-nine specimens (four groups) was carried out to study the fatigue behavior of CFRP straps, where two types of pins, titanium alloy pin and CFRP pin, and two loading frequencies, 10 Hz and 15 Hz, were used. The number of cycles to failure, displacement, fatigue failure strain, outside surface temperature at the vertex of specimen, and scanning electron microscope (SEM) photographs were recorded and analyzed to investigate the fatigue behavior of CFRP straps. The experiment results show that the temperature development at the vertex of the CFRP strap varies obviously if different pins are used due to the different friction coefficients. In addition, the fatigue life of CFRP straps decreases significantly with the increase in loading rate for the titanium pin, while it only reduces slightly with the increase in loading rate for the CFRP pin.

## 1. Introduction

For through and half-through arch bridges, hangers are the critical load-transfer components to connect bridge deck and arch ribs. However, the mechanical properties of hangers made of steel wires degrade greatly due to corrosion in coastal environment. Since the 1960s, there have been several accidents involving the rupture of bridge hangers induced by wire corrosion. Many researchers have studied the failure mechanism, preventive measures and reinforcement methods of hangers [1,2,3]. For example, to avoid the rapture of in-service cable, the corroded hangers can be replaced according to regular visual inspections. In several codes, the steel hangers have to be replaced after servicing for 20 years. However, the criteria to determine whether it should be replaced has not been well established, and most of the decisions of replacement are usually based on engineering experience.

The CFRP composites can be regarded as an ideal alternative to the steel cable. Compared to normal steel material, CFRP composites have higher tensile strength to weight ratio, higher elastic modulus to weight ratio, better corrosion resistance, and anti-fatigue capability [4,5,6,7,8]. Since the concept of a cable-stayed bridge with CFRP cables was first developed by Meier in 1987, CFRP cables have been successfully employed in a few bridges such as the Storkbrücke bridge (Switzerland, 1996), Herning footbridge (Denmark, 1999), Laroin footbridge (France, 2002), Penobscot Narrows bridge (USA, 2006), Jiangsu University foot bridge (China, 2006), and Stuttgart Stadtbahn bridge (Germany, 2020). Gui-hua Xie et al. [9] conclude that the usage of CFRP cables to replace steel cables can increase the fundamental bending frequency and torsion frequency and improve the resistance of long-span bridges against wind-induced vibration by comparing the simulation results of an existing 648 m long cable-stayed bridge with steel cables and CFRP cables in terms of static and dynamic behaviors.

The studies and research above show that the great performance of CFRP cable makes CFRP material possible to be used for tensile component in hangers. However, connection and anchoring become a key problem in the application due to its poor shear performance [10]. Schmidt et al. [11] investigated the bonded and non-bonded FRP tendon system, showing that there are concerns on how to achieve reliable anchorage due to brittleness of the tendons, low strength perpendicular to the fiber direction and insufficient stress transfer in anchorage/tendon interface. The performance of present anchorage has been investigated to improve the layout, and some novel anchorage cable system has also been proposed [12,13,14,15,16,17]. Winistoerfer et al. [18,19] studied the pin-loaded looped CFRP strap and suggested it can be used as practical tensile elements since the load can transfer through the pins without the requirement of additional anchoring systems. Lees et al. [20] and Hoult et al. [21,22] conducted experiments to analyze the enhancement of shear capacity of reinforced concrete due to CFRP strap. Fan et al. [23,24] investigated the distribution of stress, failure mechanism and bearing efficiency by finite element (FE) models. Nowadays, pin-loaded CFRP straps are widely implemented as rigging systems in the sailing industry and particularly in racing sailboats [25]. They have also been used in the crane industry as an alternative to steel pendant links in crawler cranes, because of better lifting capacities and easier self-erecting assembly [26]. Moreover, pin-loaded CFRP straps have been successfully used as tendons in the bowstring arch pedestrian bridge at the Swiss Federal Laboratories for Materials Testing and Research, Empa, Switzerland [8]. Due to good resistance to fatigue, the layout of carbon hangers can be designed with less consideration of the limit of fatigue stress range, so that the selection of the cable can be entirely based on the criteria of utilization in tension. As a result, the required CFRP hanger cross-sectional area can be reduced to one quarter that of the steel hanger.

Recently, Baschnagel et al. [27] reported the fretting fatigue test on pin-loaded CFRP straps. A significant temperature increase was observed at the beginning, and the peak value was found on the outside surface of the straps at the vertex area. Then, the temperature decreased slightly and increased again until fatigue failure of the strap. They [28] further carried out a fatigue test where two types of straps were tested at a loading frequency of 10 Hz, and they found that a sacrificial CFRP ply could help to slow the temperature increase in the straps and improve the fatigue performance. Therefore, it is important to understand the temperature effect on fatigue and fracture behavior of CFRP straps.

The fatigue and fracture behavior of CFRP straps considering the temperature effect has been investigated in this study. Firstly, the tensile testing of four CFRP strap specimens was conducted to investigate the fracture behavior of CFRP strap, and three stages were observed, including delamination, cracking, and brittle rupture. Then, a fatigue test of thirty-nine specimens in four groups was carried out to study the fatigue behavior of CFRP straps, where two types of pins, Titanium pin and CFRP pin, and two loading frequencies, 10 Hz and 15 Hz were used. The number of cycles to failure, displacement, fatigue failure strain, outside surface temperature at the specimen vertex, and SEM photos were recorded and analyzed to investigate the fatigue behavior of CFRP straps.

The following text is organized as follows. Section 2 presents the specimen details, experimental setup and test procedure; Section 3 shows the tensile testing results and fatigue testing results; Section 4 summarizes the conclusions and findings.

## 2. Materials and Methods

### 2.1. Specimens Details

**Figure 1:**
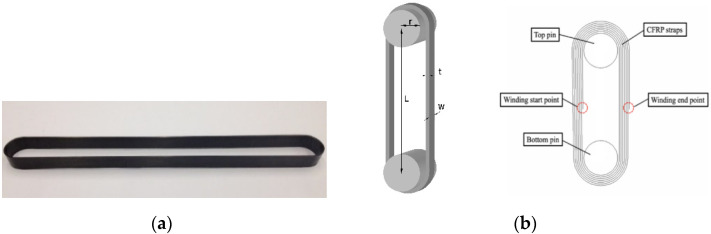
(**a**) CFRP prepreg straps; (**b**) configuration of CFRP Straps (*r* is the radius of the pins, and *t* is the thickness of the straps).

The specimens used in the experiment were CFRP prepreg straps fabricated by Carbo-Link AG of Switzerland, consisting of IMS60 carbon fibers produced by Toho Tenax Europe GmbH (Wuppertal, Germany) and XB3515/Aradur 5021 epoxy resin matrix produced by Huntsman Advanced Materials GmbH (Basel, Switzerland). The harder was XB3515/Aradur 5021 made by Huntsman Advanced Materials GmbH, Basel, Switzerland. Figure 1 shows a typical specimen. A total of forty-three specimens were prepared: four of them were used in tensile testing and the remaining thirty-nine were used in the fatigue testing. For each specimen, the symmetrical winding resulted in an overlap of the tape of approximately 1.2 mm on a seven-layer side and 1.02 mm on a six-layer side. The length between two pin centers (*L*) was about 250 mm and the width of the straps (*w*) was 12 mm. Two types of pins were used in the test; one is Titanium alloy pin and the other is CFRP pin. The pin is a cylinder with no groove. The radius and length of a pin were 10 mm and 40 mm, respectively. The material properties of CFRP straps provided by the manufacturer are shown in Table 1.

### 2.2. Experimental Setup and Test Procedure

**Figure 2:**
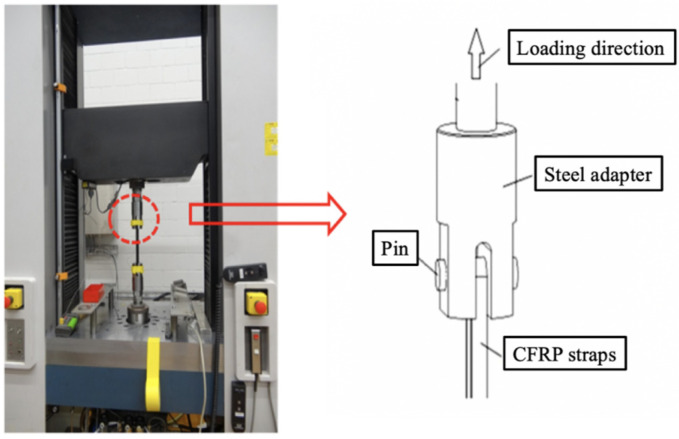
Experiment setup.

The tensile testing was conducted on a hydraulic universal tester (Instron 1251), as shown in Figure 2. The two pins were fixed in the steel adapters to transfer loading to the CFRP strap. During the test, the lower end of the strap did not move, while the upper end moved with pin and steel adapter, since the load was applied under displacement control. The loading rate was at 2 mm/min, and the loading direction was upwards, which was consistent with the direction of the fiber in the CFRP strap. The hydraulic universal tester kept loading until failure of the CFRP strap. During the loading process, the displacement and corresponding load value were automatically recorded. Four specimens were used in the tensile testing, namely, ST-1, ST-2, ST-3 and ST-4, respectively.

The fatigue testing was also carried out to investigate the fatigue performance of CFRP straps. The loading machine, the pins and steel adapters, and the specimens were the same as those in the tensile testing. The maximum stress was 1000 MPa and the corresponding load was 2600 N; the ratio of minimum to maximum stress was 0.1 and the loading frequencies were 10 Hz and 15 Hz. It should be noted that 10 Hz represents a realistic loading frequency observed in the previous study [28]. Thirty-nine specimens were used in the fatigue testing, which can be seen in Table 2. FA-Ti10-X and FA-Ti15-X indicated the specimens were loaded at rates of 10 Hz and 15 Hz with titanium alloy pins; FA-CFRP10-X and FA-CFRP15-X indicated the specimens were loaded at a rate of 10 Hz and 15 Hz with CFRP pins. The type K thermocouple was attached on the outside surface of the specimen vertex to measure the temperature development in the critical area. Except for temperature, displacement, strain, and number of load cycles to fatigue were also measured during the test.

## 3. Results

### 3.1. The Tensile Testing Results

The load-carrying capacity of the CFRP strap was first estimated through the tensile testing. In particular, the damage mode, stress–strain curve and ultimate strength have been presented.

#### 3.1.1. Damage Mode

**Figure 3:**
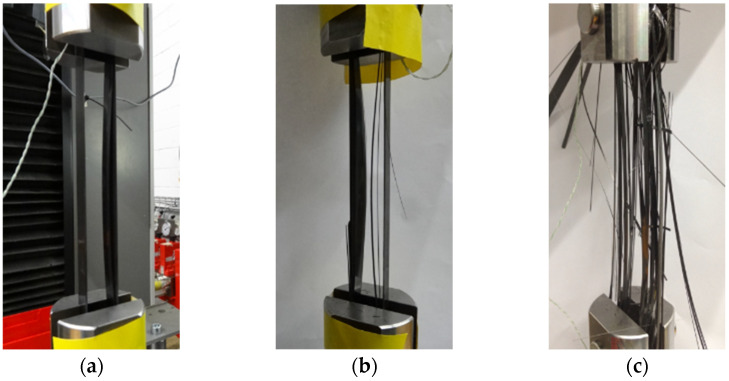
Failure process of the CFRP strap. (**a**) Delamination; (**b**) Cracking; (**c**) Brittle rupture.

**Figure 4:**
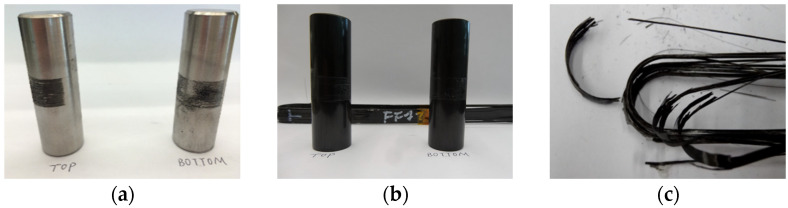
(**a**) Titanium alloy pins (**b**) CFRP pins and (**c**) damaged CFRP strap after failure.

Figure 3 shows the failure process of the CFRP strap during the tensile testing. As the loading increased, the delamination of CFRP strap was observed at the intersection between straight and curved segment and then extended to the curved segment, because the shear stress between layers exceeded the ultimate strength of epoxy resin. Then, longitudinal cracking initiated and propagated soon, which resulted in that each layer of CFRP worked independently due to the loss of adhesion between layers. Finally, brittle fracture of the CFRP strap occurred between the straight and curved segment. Figure 4 shows the damaged parts of the CFRP strap and the pins after failure. The titanium alloy pins were almost intact while abrasive wear can be observed on the CFRP pins.

#### 3.1.2. Stress–Strain Curve and Ultimate Tensile Strength

**Figure 5:**
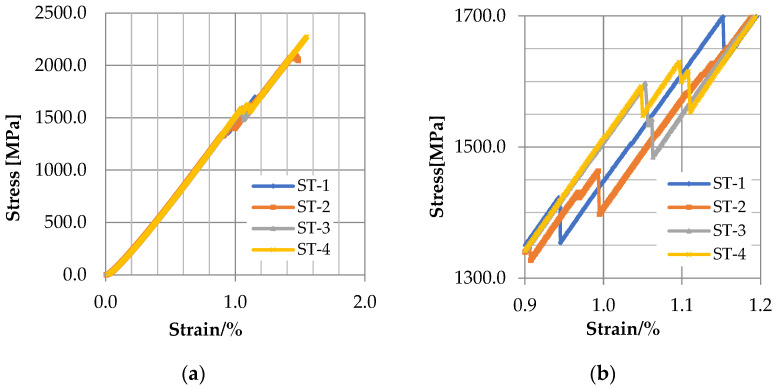
(**a**) Stress–strain curves of the four specimens (**b**) enlarged view.

The stress–strain curves of the four specimens are shown in Figure 5. Generally, they look quite similar, and there is no obvious discrepancy between curves. For each curve, the stress is in proportion to the strain, and the gradient matches the elastic modulus provided by the manufacturer. A sudden drop can be observed on the strain–stress curve when the strain is nearly 1%, indicating the occurrence of delamination, and the corresponding stress is between 1422.85 to 1615.49 MPa, as shown in Table 3. As the stress further increases to about 2000 MPa, the strain reaches nearly 1.5%, corresponding to the brittle fracture of CFRP strap. Table 3 also shows that the ultimate tensile strength of the specimens is in the range of 1924–2275 MPa and the average ultimate tensile strength is 2078.39 MPa, which is about 80% of that value of straight CFRP straps provided by the manufacturer. This is due to the stress concentration at the intersection between the curved part and the straight part. In fact, the specimen quickly developed to fracture failure after the delamination was observed in the test. Therefore, the delamination of CFRP straps can be regarded as a sign of failure, which should be avoided to ensure the overall safety and stability of the CFRP straps.

### 3.2. The Fatigue Testing Results

#### 3.2.1. Displacement Development

**Figure 6:**
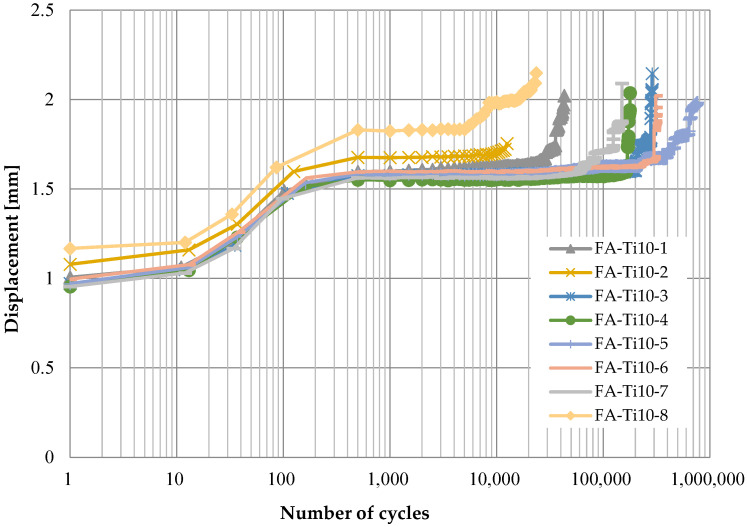
Displacement development of specimens with respect to number of cycles (titanium alloy pin, 10 Hz).

**Figure 7:**
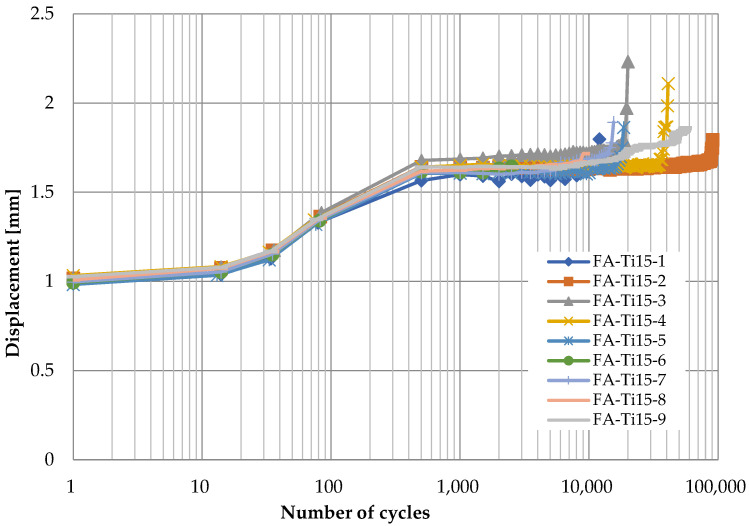
Displacement development of specimens with respect to number of cycles (titanium alloy pin, 15 Hz).

**Figure 8:**
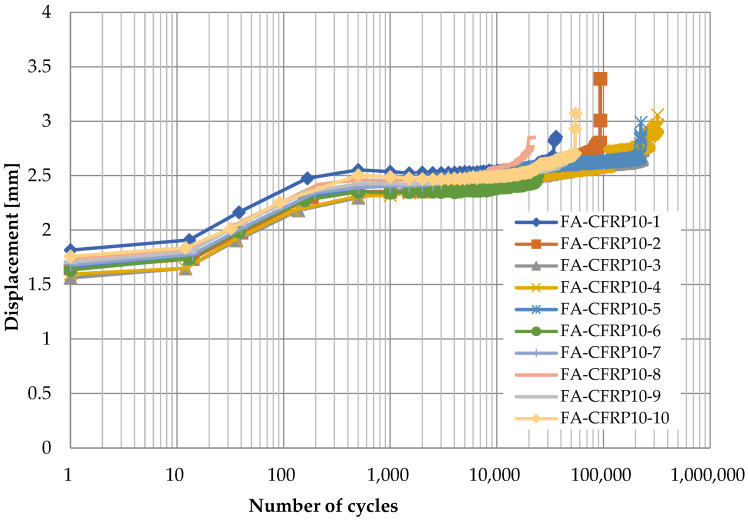
Displacement development of specimens with respect to number of cycles (CFRP pin, 10 Hz).

**Figure 9:**
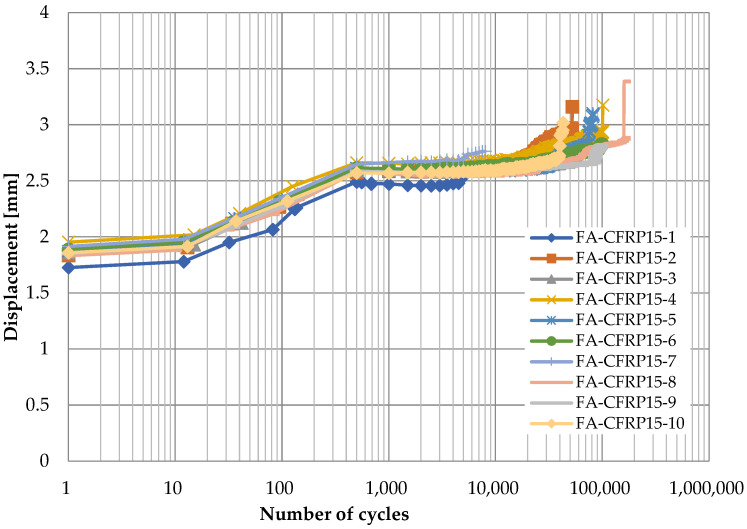
Displacement development of specimens with respect to number of cycles (CFRP pin, 15 Hz).

**Figure 10:**
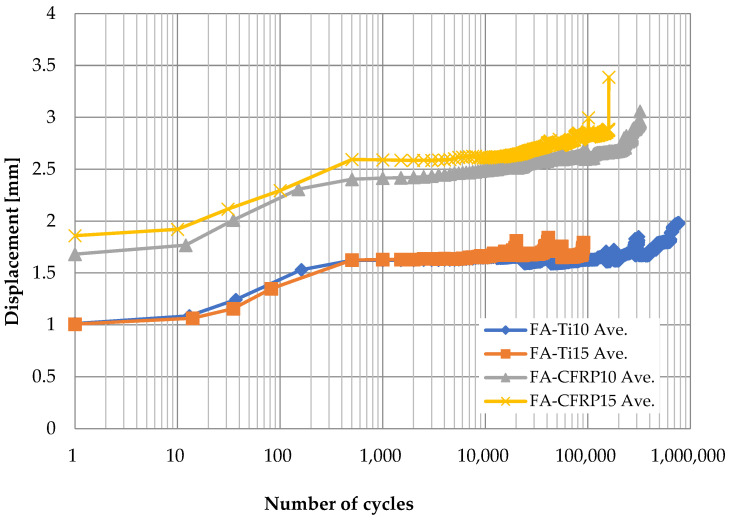
Average displacement of specimens with respect to number of cycles.

Figure 6, Figure 7, Figure 8 and Figure 9 show the displacement of specimens with respect to the number of cycles for different pins and different loading rates, which look quite similar. For better illustration, the average displacements of specimens for various conditions are shown in Figure 10. Generally, it is observed the displacement begin to increase after 10 cycles until the applied load reaches the maximum value of 24 kN after 1000 cycles. Then, the displacement keeps stable (1.8 mm for titanium alloy pin and 2.5 mm for CFRP pin) for a long time during which the number of cycles increases from 10^3^ to 10^5^. As the number of cycles further increases, a sudden jump on the curve of displacement development in Figure 6, Figure 7, Figure 8 and Figure 9 can be observed, indicating the delamination of the CFRP strap and the subsequent failure of the CFRP strap. From Figure 10, it is also observed that the displacement of CFRP straps when CFRP pins were used is nearly 0.8 mm greater than that when titanium pins were used, regardless of loading rate and number of cycles, because the CFRP pins had larger deflection than the titanium alloy pins during the fatigue test.

#### 3.2.2. Failure Strain

**Figure 11:**
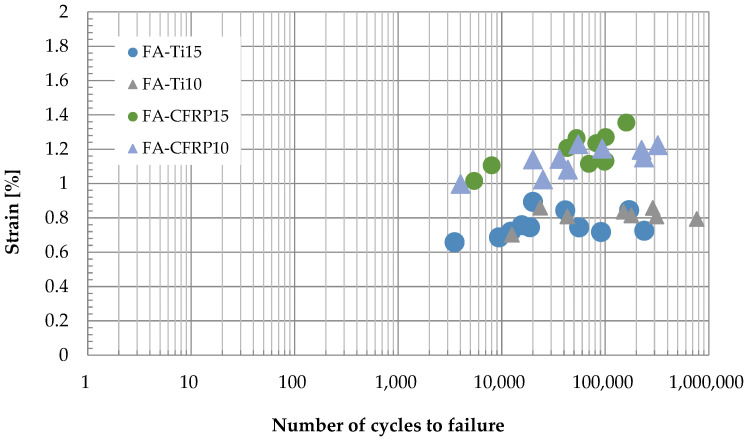
Fatigue life diagram of the pin-loaded straps.

Figure 11 shows the strain at failure and the corresponding number of cycles to failure for specimens in various conditions. When the titanium alloy pins were used in the test and the loading rate was 10 Hz, it is seen from Figure 11 that the strain at failure is in the range of 0.70% to 0.86% and the number of cycles to failure varies from 3.5 × 10^3^ to 2.38 × 10^4^; as the loading rate increased to 15 Hz, the strain at failure is in the range of 0.66% to 0.89%, and the number of cycles to failure varies from 1.25 × 10^4^ to 7.65 × 10^5^. When the CFRP pins were used in the test, it is observed from Figure 11 that the strain at failure is in the range of 1.00% to 1.22% (loading rate 10 Hz) and 1.02% to 1.35% (loading rate 15 Hz), and the number of cycles to failure varies from 4 × 10^3^ to 3.22 × 10^5^ (loading rate 10 Hz) and 5.4 × 10^3^ to 1.6 × 10^5^ (loading rate 15 Hz). Generally, the strain at failure for CFRP pins is about 0.4% larger than that for titanium alloy pins due to the greater deflection of CFRP pins, which coincides with the observation in Figure 10.

#### 3.2.3. Temperature Development

The cyclic load in the fatigue testing caused friction between the CFRP strap and the pins, and the accumulated heat cannot transfer quickly due to the low thermal conductivity coefficient of CFRP and air, making the temperature of interface between CFRP strap and pin increase significantly. Therefore, the temperature development at the vertex of the CFRP strap was monitored during the test.

**Figure 12:**
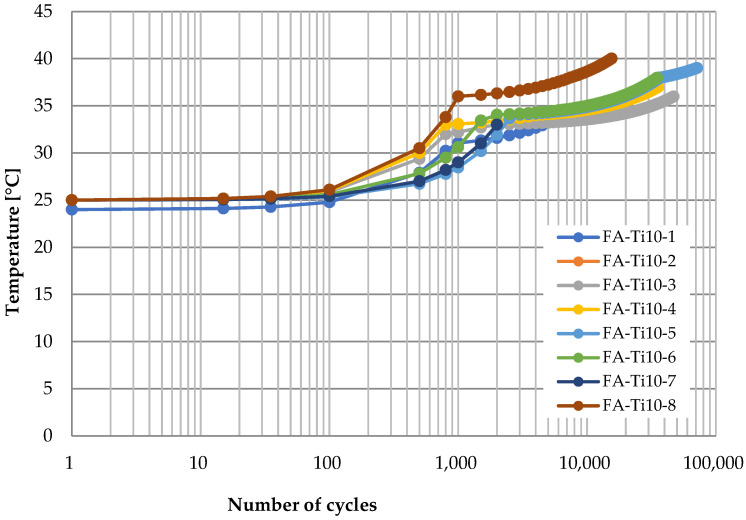
Temperature development at the vertex of CFRP straps (titanium alloy pin, 10 Hz).

Figure 12 shows the time history of temperature at the vertex of CFRP strap when the titanium alloy pins were adopted and the loading rate was 10 Hz in the fatigue testing. Generally, there are three stages: first, the temperature almost keeps constant at the ambient temperature with the logarithm of the number of cycles (0–10^2^ cycles); second, it raises gradually to about 35 °C as the number of cycles increases (10^2^–10^3^ cycles); third, it continues to increase but the increasing rate decreases obviously (10^3^–10^5^ cycles), and it can be nearly 40 °C until failure of the CFRP strap. At the first stage, the heat induced by friction accumulated slowly since the titanium alloy has a great thermal conductivity coefficient, and the temperature at the inside surface of the vertex of the CFRP strap increased slightly. Then, the temperature gradient formed, and the heat transfer started from the inside surface to the outside surface. Since the temperature gradient was quite small at this stage, heat transfer speed was quite limited, and therefore, the temperature at the outside surface of the vertex of CFRP strap still did not change. At the second stage, the temperature at the outside surface started to increase due to the temperature gradient between the inside surface and outside surface. However, the temperature gradient between the outside surface and the surrounding environment was negligible; therefore, the heat transfer from the CFRP strap to the surrounding environment was limited. At the third stage, as the temperature at the outside surface increased to about 35 °C, the heat transfer from the CFRP strap to the surrounding environment started, and therefore, although the temperature at the outside surface of the vertex of CFRP strap still increased, the increasing rate was less than that in the second stage.

**Figure 13:**
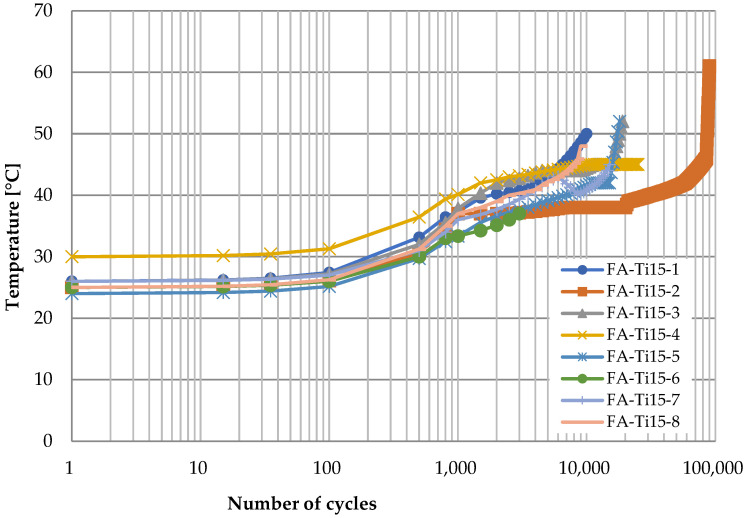
Temperature development at the vertex of CFRP straps (titanium alloy pin, 15 Hz).

Figure 13 shows the temperature development at the CFRP strap vertex when the titanium alloy pins were used and the loading rate increased to 15 Hz. The temperature development looks quite similar. However, the temperature increasing rate at the second and the third stages is slightly greater than that in Figure 12, because the higher loading rate made greater heat induced by friction in the fatigue test. In addition, it is observed from Figure 13 that the temperature increases drastically before the failure of the CFRP strap. There are two possible reasons. On one hand, the heat transfer from the CFRP strap to the air is quite limited due to the low thermal conductivity coefficient of air. Hence, only a small portion of the heat induced by friction can transfer to the surrounding environment. On the other hand, the fibers near the inside surface are damaged and the matrix is deteriorated, making the contact surface less smooth. That is, the friction coefficient increases and the heat induced by the friction increases accordingly.

**Figure 14:**
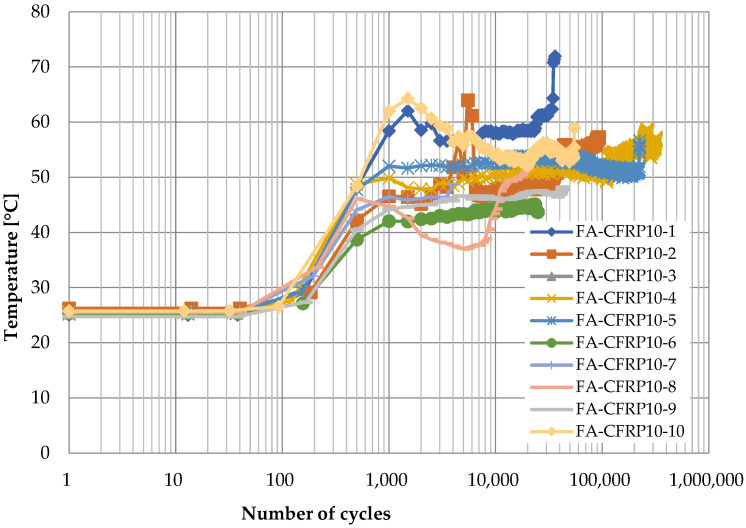
Temperature development at the vertex of CFRP straps (Titanium alloy pin, 10 Hz).

**Figure 15:**
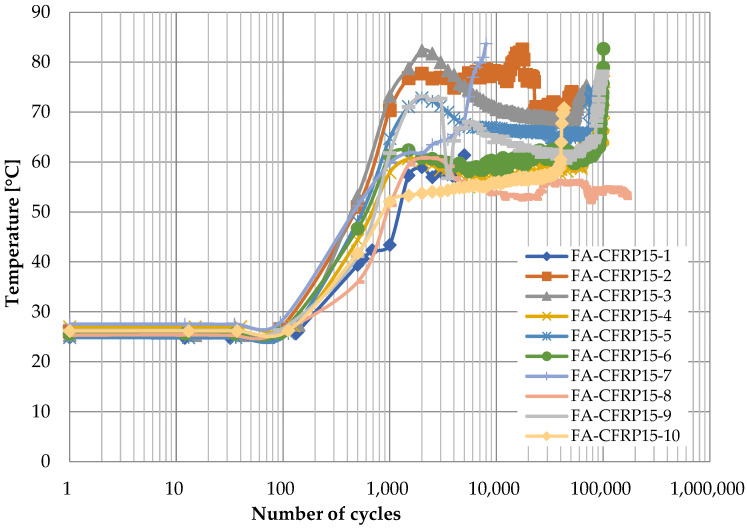
Temperature development at the vertex of CFRP straps (titanium alloy pin, 15 Hz).

Figure 14 and Figure 15 show the time history of temperature at the vertex of the CFRP strap when the CFRP pins were adopted and the loading rates were 10 Hz and 15 Hz, respectively. Similarly, there are also three stages. The temperature remains constant at ambient temperature at the first stage, and it increases gradually with the number of cycles at the second stage. However, it can reach 40 to 60 °C (Figure 14, loading rate of 10 Hz) or 60 to 80 °C (Figure 15, loading rate of 15 Hz) at the end of this stage, which is much higher than that when the titanium alloy pins were used, because CFRP has a much lower thermal conductivity coefficient of CFRP than titanium, and hence, the heat induced by friction can hardly transfer to the CFRP pin. Different from Figure 12 and Figure 13, the temperature does not further increase at the third stage; instead, it decreases slightly as the number of cycles increases for some specimens. This is because the temperature gradient between the CFRP strap and the air was greater, and therefore, the heat could transfer more quickly from the CFRP strap to the surrounding environment. Similarly, a sudden increase in temperature can be observed before the failure of CFRP straps.

**Figure 16:**
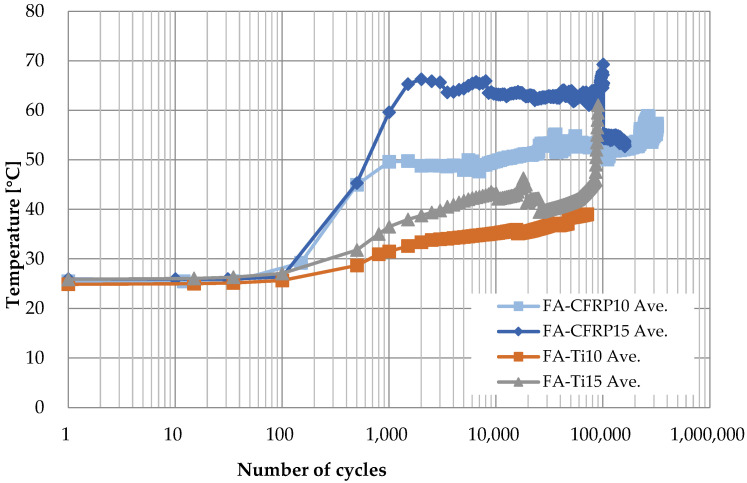
Surface average temperature at the top of the strap for four situations.

For better comparison, the curves of average temperature of different specimens for the four cases are shown in Figure 16, where the three stages for each case can be identified clearly. On one hand, the temperature is higher for CFRP pins because the CFRP has lower thermal conductivity coefficient; on the other hand, when the material of pins is the same, the temperature is higher if the loading rate is higher due to greater heat induced by friction between the pin and the CFRP strap.

#### 3.2.4. Fatigue Life

**Figure 17:**
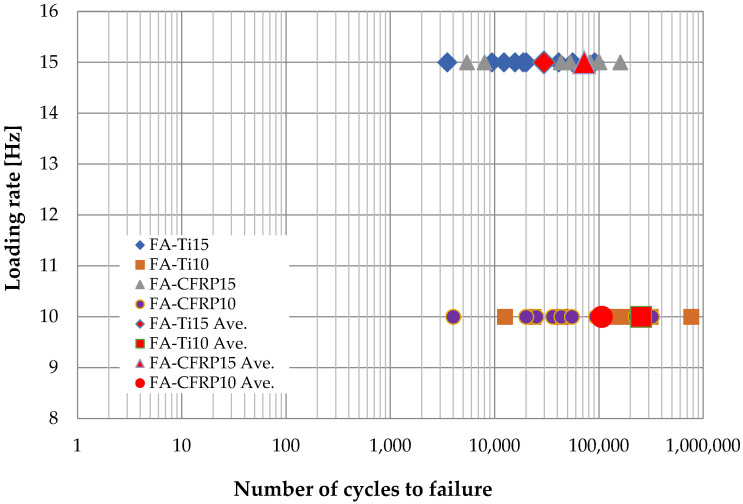
Fatigue life of specimens.

Based on the result of tensile testing, the fatigue load was set in the range of 2.4 to 24 kN to avoid delamination, where the ratio of minimum and maximum stress was 0.1. The number of cycles to fatigue of all CFRP straps is shown in Figure 17. When the CFRP pins are used, the fatigue life of CFRP straps decreases slightly from 1.1 × 10^5^ to 7.2 × 10^4^ as the loading rate increases from 10 to 15 Hz. When the titanium alloy pins are used, the fatigue life of CFRP straps decreases significantly from 2.5 × 10^5^ to 3.0 × 10^4^ as the loading rate increases from 10 to 15 Hz. Therefore, the effect of temperature increase induced by friction on the fatigue CFRP strap is slight if CFRP pins are used and significant if titanium alloy pins are used.

#### 3.2.5. Scanning Electron Microscopy (SEM) Analysis

**Figure 18:**
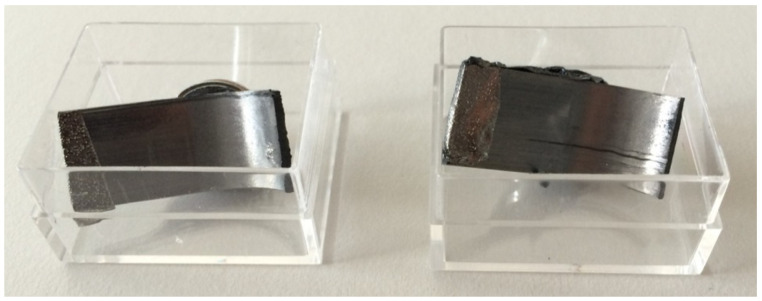
Test pieces prepared for the SEM analysis (FA-Ti15-7).

SEM analysis was conducted to investigate the fretting behavior of the contact surface between the CFRP strap and pin. Two pieces of the CFRP strap shown in Figure 18 were cut from FA-Ti15-7. The fatigue test was not interrupted during loading, and it was terminated at failure only. The two pieces were collected after the fatigue test, spray-cleaned by argon and finally gold-sputtered for 3 min in a vacuum with nitrogen. One was the curved part contacting to the lower pin and the other was the curved part contacting to the upper pin. The contact surfaces of the test pieces were examined by SEM (NovaNanoSEM 230).

**Figure 19:**
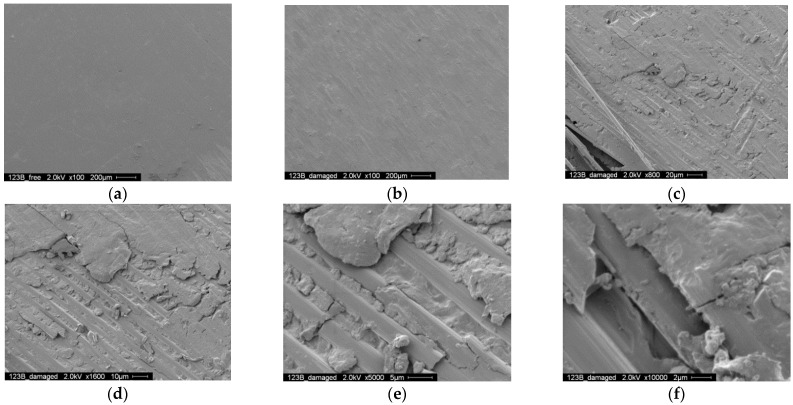
SEM photographs of the contact surface (fretted area near lower pin, 14,000 cycles): (**a**) 200 μm, intact (**b**) 200 μm, damaged (**c**) 20 μm (**d**) 10 μm (**e**) 5 μm (**f**) 2 μm.

Figure 19 shows the SEM photographs of the contact surface of the curved part contacting to the lower pin. Figure 19a,b show the intact and damaged area on the contact surface, and it can be observed that the intact area is still smooth while the damaged area is uneven due to the broken fibers. Figure 19c–f show the SEM photographs with higher magnification, where matrix deterioration and broken fibers can be observed clearly, indicating the damage of the CFRP strap.

**Figure 20:**
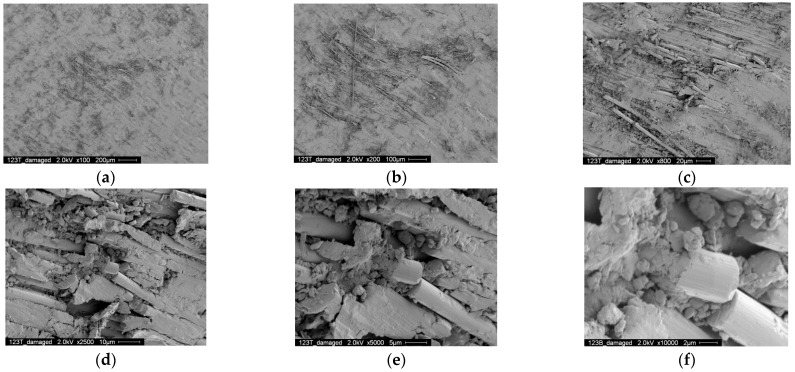
SEM photographs of the contact surface (fretted area near upper pin, 14,000 cycles) (**a**) 200 μm (**b**) 100 μm (**c**) 20 μm (**d**) 10 μm (**e**) 5 μm (**f**) 2 μm.

Figure 20 shows the SEM photographs of the contact surface of the curved part contacting the upper pin. Figure 20a shows the overall view of the damaged area on the contact surface, and Figure 20b–f show the SEM photographs with higher magnification. Similar to Figure 19, matrix deterioration and broken fibers can be observed, but the fretting damage is more severe. In fact, when such severe fretting damage occurs, it increases the surface roughness of the CRFP strap. Therefore, the heat induced by friction increases more rapidly, creating a significant temperature increase in the outside surface of the CFRP strap vertex before failure.

## 4. Conclusions

The static tensile behavior and fatigue behavior of pin-loaded CFRP straps has been investigated in this study, where the temperature effect has been considered. Two different pins, titanium alloy pin and CFRP pin, and two different loading frequencies, 15 Hz and 10 Hz, were adopted in the test. The damage mode and stress–strain curve in the tensile testing have been recorded, and the number of cycles to failure, displacement, fatigue failure strain, outside surface temperature at the end of specimen, and scanning electron microscope (SEM) photos were monitored in the fatigue testing. There are the findings:

1. At the intersection between the straight and curved segments of CFRP strap, there is a significant stress concentration, resulting in a nearly 20% reduction in the ultimate tensile strength of the straight CFRP strap compared with the straight strip. In addition, the delamination of the CFRP strap generally occurs when the stress reaches about 70% of the ultimate tensile strength. After the delamination, the CFRP strap fails soon; therefore, the delamination can be considered as a sign of failure.
2. The displacement of the CFRP strap is nearly 0.8 mm larger if CFRP pins are used instead of titanium pins, regardless of loading rate and number of cycles, because the CFRP pins have greater deflection than the titanium alloy pins during the fatigue testing.
3. In the fatigue testing, the temperature development of the outside surface of the CFRP vertex has three stages: first, the temperature remains constant when the number of cycles is less than 100; second, the temperature increases gradually when the number of cycles increases from 100 to 1000; thirdly, the temperature keeps increasing with a lower increasing rate for titanium alloy pins but slightly decreases for CFRP pins. As the number of cycles further increases, the temperature increases significantly because the damaged fibers and matrix increase the surface roughness, which further magnifies the friction. Hence, it is necessary to enhance the performance of the CFRP strap, and enriching resins with carbon nanotubes may be investigated in the future [29,30].
4. The effect of temperature increase induced by friction on the fatigue CFRP strap is slight if CFRP pins are used and significant if titanium alloy pins are used.

## Figures and Tables

**Table 1:** Material parameters of carbon fiber, epoxy resin and CFRP strap.

Properties	Carbon Fiber	Epoxy Resin	CFRP Strap
Ultimate strength (MPa)	5600	120~140	2567 ± 58
Modulus of elasticity (GPa)	290	2.9~3.1	168 ± 6.6
Ultimate tensile strain (%)	/	/	1.52 ± 0.23
Carbon fiber volume content (%)	/	/	62 ± 2
Thickness single layer (mm)			0.17
Width (mm)			12

**Table 2:** Specimen ID in the fatigue testing.

Specimen ID	Pin Material	Loading Frequency (Hz)	Thickness Seven Layers/Six Layers (mm)	Width (mm)
FA-Ti10-1~8	Titanium alloy	15	1.2/1.02	12
FA-Ti15-1~11	Titanium alloy	15	1.2/1.02	12
FA-CFRP10-1~10	CFRP	10	1.2/1.02	12
FA-CFRP15-1~10	CFRP	10	1.2/1.02	12

**Table 3:** The results of the CFRP straps static tensile test.

Specimen Number	Ultimate Tensile Stress/MPa	Strain /%	Stress at Occurrence of Delamination/MPa	Strain/%
ST-1	2067.96	1.42	1422.85	0.94
ST-2	2047.25	1.49	1463.40	0.99
ST-3	1924.03	1.33	1597.37	1.05
ST-4	2274.30	1.55	1615.49	1.11
Avg.	2078.39	1.45	1524.78	1.02

## Data Availability

Not applicable.
